# Natural Products as Potential Leads Against Coronaviruses: Could They be Encouraging Structural Models Against SARS-CoV-2?

**DOI:** 10.1007/s13659-020-00250-4

**Published:** 2020-06-11

**Authors:** Ilkay Erdogan Orhan, F. Sezer Senol Deniz

**Affiliations:** Department of Pharmacognosy, Faculty of Pharmacy, Gazi University, 06330 Ankara, Turkey

**Keywords:** Coronavirus, SARS-CoV, Medicinal plants, Natural products, Antiviral

## Abstract

New coronavirus referred to SARS-CoV-2 has caused a worldwide pandemic (COVID-19) declared by WHO. Coronavirus disease 2019 (COVID-19) is an infectious disease with severe acute respiratory syndrome caused by coronavirus-2 (SARS-CoV-2). SARS-CoV-2 is akin to SARS-CoV, which was the causative agent of severe acute respiratory syndrome (SARS) in 2002 as well as to that of Middle East respiratory syndrome (MERS) in 2012. SARS-CoV-2 has been revealed to belong to Coronaviridiae family as a member of β-coronaviruses. It has a positive-sense single-stranded RNA with the largest RNA genome. Since its genomic sequence has a notable similarity to that of SARS-CoV, antiviral drugs used to treat SARS and MERS are now being also applied for COVID-19 treatment. In order to combat SARS-CoV-2, many drug and vaccine development studies at experimental and clinical levels are currently conducted worldwide. In this sense, medicinal plants and the pure natural molecules isolated from plants have been reported to exhibit significant inhibitory antiviral activity against SARS-CoV and other types of coronaviruses. In the present review, plant extracts and natural molecules with the mentioned activity are discussed in order to give inspiration to researchers to take these molecules into consideration against SARS-CoV-2.

## Introduction

Coronaviruses (family of Coronaviridiae, order of Nidovirales) named for the crown-like spikes on their surface are described as a family consisting of enveloped, single-stranded, and positive-strand RNA viruses possessing a helical nucleocapsid. They are known to cause acute and chronic respiratory, enteric, and central nervous system diseases in animals and humans [1]. Some types of coronaviruses are known to be hosted by humans including HCoV-229E, HCoV-HKU1, and HCoV-NL-63 (α-coronoviruses), HCoV-OC43, MERS-CoV, and SARS-CoV (β-coronoviruses). On the other hand, other types of coronaviruses using animal species as host are also available such as transmissible gastroenteritis virus (TGEV) and porcine respiratory coronavirus (PRCoV) in pigs, feline infectious peritonitis virus (FIPV) and feline enteric coronavirus (FeCoV) in cats, bovine respiratory coronavirus (BCoV) in cows, infectious bronchitis virus (IBV) in chickens and birds also known as avian coronavirus, mouse hepatitis virus (MHV), etc. They are generally classified under three groups as group I (TGEV, PEDV, FIPV, PRCV, and CCoV), group II (MHV, BCoV, and HCoV-OC43), and group III (TCoV and IBV) [2]. SARS-CoV was reported to cross-react with some members of group I coronavirus antibodies [3]. It should be also noted that, in addition to α- and β-coronoviruses, δ- (NHCoV HKU-19, WiCoV HKU-20, PorCoV HKU-15, CmCoV HKU21), and γ-coronoviruses (TCoV, BWCoV SW1, IBC-partridge) are present in the classification.

A coronavirus-based disease was firstly diagnosed in 1931, while the first coronavirus (HCoV-229E) was isolated from humans in 1965. Only HCoV-229E and HCoV-OC43 were known until SARS-CoV, being a member of the subgenus Sarbecovirus, which was realized as severe acute respiratory syndrome (SARS). It was defined a contagious and often fatal respiratory illness, firstly reported in Guandong province, China, in November 2002 with 11% of mortality. After that, Middle East Respiratory Syndrom (MERS-CoV) epidemic caused by the member of Merbecovirus subgenus of β-coronoviruses was seen in Saudi Arabia in 2012 with 34% of mortality rate, which was said to transmit from camels to humans. Coronavirus infectious disease (COVID-19) named by WHO, appeared firstly in Wuhan (China) in December, 2019 is described to be an infectious disease particularly affecting respiratory tract. It was caused by a newly discovered coronavirus (SARS-CoV-2) and has become a worldwide pandemic with the widespread emergence. World Health Organization (WHO) announced COVID-19 as international pandemic on January 30th, 2020. As May 31st, 2020, totally 5,819,962 confirmed COVID-19 cases and 362,876 deaths in the world (215 countries in total) have been updated by WHO official statistics.

SARS-CoV-2, also referred as HCoV-19 as a member of the genus β-coronavirus and the subgenus Sarbecovirus, is said to transmit to humans by zoonotic transfer through a precursor virus from insectivorous bats (*Rhinolophus affinis*) or pangolins (*Manis javanica*). The new coronavirus causes mainly severe acute respiratory syndrome (SARS) interacting through its high binding affinity to angiotensin-converting enzyme-2 (ACE-2) receptors as well as trans membrane serine protease (TMPPRS) co-receptors for S protein priming [4, 5].

Not only SARS, but also MERS-CoV is a zoonotic virus, both of which contain polycistronic plus-stranded RNA that are known to be the largest RNA genomes. Coronaviruses ranging from 26 to 32 kilobases in length are known to possess three major proteins consisting of the very large (200 K) glycoprotein S (spike protein) located in the viral envelope, a transmembrane glycoprotein (M protein), and the internal phosphorylated nucleocapsid protein (N protein) [6]. SARS-CoV-2 has been revealed to be closely related to both SARS-CoV and MERS-CoV. It has been reported to share approximately 70–80% of its genome with that of SARS-CoV. The recent SARS-CoV-2 genome sequencing pointed out to a different genome composition in relation to its relative βCoV strains isolated from human (SARS-CoV) [7, 8].

Despite of the scarcity of our information on SARS-CoV-2, a number of known antiviral drugs (lopinavir/ritonavir, darunavir/umifenovir, oseltamivir, favipiravir, remdesivir, etc.) used against SARS-CoV as well as some other drugs such as chloroquine, hydroxychloroquine, azithromycin, tocilizumab, interpheron-β, etc. have been implemented for the treatment of COVID-19 patients or in clinical trials [9–12]. Many studies at laboratory and clinical levels are extensively going on to discover an effective medication or therapy to beat SARS-CoV-2.

Taking the information into account that many natural products have been fruitful leads and molecular frameworks for developing new clinical drugs since a long time, we thought that medicinal plants and their secondary metabolites with anti-coronavirus activity may shed a light on COVID-19 drug discovery and development and can inspire the researchers working on this subject. Therefore, aim of the current review was to search medicinal plants and natural molecules with antiviral activity, which have been already reported to be effective against SARS-CoV and some other types of coronaviruses. Since this is a worldwide hot topic, the plant extracts and natural compounds obtained from plants with SARS-CoV inhibitory activity through various mechanisms have been searched extensively in PubMed database using the keywords “plants and coronavirus”. The relevant literatures were divided into two main parts as plant extracts and natural molecules (Table 1).

**Table 1:** Examples of some promising natural molecules with coronavirus inhibitory activity

Compound	Chemical group	IC_50_/EC_50_ values	Coronavirus type targeted	References
Glycyrrhizin	Saponin	EC_50_ = 364.5 μM	SARS-CoV	[26]
Saikosaponin B2	Saponin	EC_50_ = 1.7 ± 0.1 mmol/L	SARS-CoV	[29]
Saikosaponin A	Saponin	EC_50_ = 8.6 ± 0.3 mmol/L	SARS-CoV	
Tetra-O-galloyl-β-D-glucose	Polyphenol	EC_50_ = 4.5 μM	SARS-CoV	[30]
Luteolin	Flavonoid	EC_50_ = 10.6 μM	SARS-CoV	
Sinigrin	Polyphenol	IC_50_ = 217 μM	SARS-CoV	[31]
β-Sitosterol	Phytosterol	IC_50_ = 1210 μM	SARS-CoV	
Hesperetin	Flavonoid	IC_50_ = 8.3 μM	SARS-CoV	
Amentoflavone	Flavonoid	IC_50_ = 8.3 μM	SARS-CoV	[33]
Luteolin	Flavonoid	IC_50_ = 20.2 μM	SARS-CoV	
Quercetin	Flavonoid	IC_50_ = 23.8 μM	SARS-CoV	
Apigenin	Flavonoid	IC_50_ = 280.8 μM	SARS-CoV	
Isobavachalcone	Flavonoid	IC_50_ = 7.3 ± 0.8 μM	SARS-CoV	[34]
Psoralidin	Flavonoid	IC_50_ = 4.2 ± 1.0 μM	SARS-CoV	
Tomentin A	Flavonoid	IC_50_ = 6.2 ± 0.04 μM	SARS-CoV	[35]
Tomentin B	Flavonoid	IC_50_ = 6.1 ± 0.02 μM	SARS-CoV	
Tomentin E	Flavonoid	IC_50_ = 5.0 ± 0.06 μM	SARS-CoV	
3′-O-Methyldiplacol	Flavonoid	IC_50_ = 9.5 ± 0.10 μM	SARS-CoV	
Isoliquiritigenin	Flavonoid	IC_50_ = 61.9 ± 11.0 μM	SARS-CoV	[37]
Quercetin	Flavonoid	IC_50_ = 52.7 ± 4.1 μM	SARS-CoV	
Kaempferol	Flavonoid	IC_50_ = 116.3 ± 7.1 μM	SARS-CoV	
Kazinol F	Flavonoid	IC_50_ = 43.3 ± 10.4 μM	SARS-CoV	
Broussochalcone B	Flavonoid	IC_50_ = 57.8 ± 0.5 μM	SARS-CoV	
Papyriflavonol A	Flavonoid	IC_50_ = 103.6 ± 17.4 μM	SARS-CoV	
Terrestrimine	Cinnamic amide	IC_50_ = 15.8 ± 0.6 μM	SARS-CoV	[36]
Blancoxanthone	Xanthone	EC_50_ = 3 μg/ml	HCoV 229E	[38]
Pyranojacareubin	Xanthone	EC_50_ = 15 μg/ml	HCoV 229E	
Lycorine	Alkaloid	EC_50_ = 15.7 IU/ml	SARS-CoV	[41]
Tylophorine	Alkaloid	EC_50_ = 58 ± 4 nM	TGEV	[42]
7-Methoxycryptopleurine	Alkaloid	EC_50_ = 20 ± 1 nM	TGEV	
Jubanine G	Alkaloid	EC_50_ = 13.41 ± 1.13 μM	PEDV	[43]
Jubanine H	Alkaloid	EC_50_ = 4.49 ± 0.67 μM	PEDV	
Nummularine B	Alkaloid	EC_50_ = 6.17 ± 0.50 μM	PEDV	
Tingenone	Triterpene	IC_50_ = 9.9 ± 0.1 μM	SARS-CoV	[33]
Iguesterin	Triterpene	IC_50_ = 2.6 ± 0.3 μM	SARS-CoV	
Pristimererin	Triterpene	IC_50_ = 5.5 ± 0.7 μM	SARS-CoV	
Dihydrotanshinone I	Diterpene	IC_50_ = 4.9 ± 1.2 μM	SARS-CoV	[45]
Cryptotanshinone	Diterpene	IC_50_ = 0.8 ± 0.2 μM	SARS-CoV	
Tanshinone IIA	Diterpene	IC_50_ = 1.6 ± 0.5 μM	SARS-CoV	
Schimperinone	Triterpene	EC_50_ = 0.28 ± 0.09 μM	PEDV	[45]
Xanthoangelol	Chalcone	IC_50_ = 11.4 ± 1.4 μM	SARS-CoV	[52]
Hirsutenone	Diarylheptanoid	IC_50_ = 3.0 ± 1.1 μM	SARS-CoV	[53]
Rubranoside	Diarylheptanoid	IC_50_ = 7.2 ± 2.2 μM	SARS-CoV	
Curcumin	Diarylheptanoid	IC_50_ = 5.7 μM	SARS-CoV	
APA	Lectin	EC_50_ = 0.45 ± 0.08 μg/ml	SARS-CoV	[54]
UDA	Lectin	EC_50_ = 1.3 ± 0.1 μg/ml	SARS-CoV	[54]

### Plant Extracts

Screening of plant extracts for their biological activity beforehand is a preferred experimental strategy to reach their bioactive molecules in the end. Our literature survey based on PubMed database indicated presence of a number of papers investigating antiviral activity of plant extracts against diverse types of coronaviruses, SARS-CoV in particular. In an early antiviral study screening 100 medicinal plants from British Columbia (Canada) against various coronaviruses, only the branch extracts of *Rosa nutkana* C. Presl and *Amelunchier alnifolia* (Nutt.) Nutt., both from Rosaceae family, were active against an enteric coronavirus of bovine origin (BCoV), which led to a complete inhibition of virus-induced cytopathogenic effect (CPE) [13]. It might also be worth to note that the branch extracts of *Potentilla arguta* Pursh. (Rosaceae) and *Sambucus racemosa* L. (Adoxaceae) were able to inhibit respiratory syncytial virus, which possesses a single-stranded RNA-like coronaviruses. An antiviral plant extract prepared from the African *Trifolium* species (Fabaceae), coded Secomet-V, was found to inhibit SARS-CoV through blocking viral entry [14]. However, active ingredient in the plant extract was not identified, where no more information was available in the paper as the extract was tested only in a coded panel. In another screening study on the South Korean medicinal plants, 22 traditionally used plants were tested against MHV-A59-infected mouse dihydrolipoamide-branched chain transacylase E2 (DBT) cells [15]. Out of these plants, *Cimicifuga racemosa* (L.) Nutt. (Ranunculaceae) rhizomes (EC_50_ = 19.4 ± 7.0 μg/ml, selectivity index (SI) = 12.3), *Melia azedarach* L. (Meliaceae) cortex (EC_50_ = 13.0 ± 1.4 μg/ml, SI = 25.6), *Coptis chinensis* Franch. (Ranunculaceae) rhizomes (EC_50_ = 2.0 ± 0.5 μg/ml, SI = 34.9), *Phellodendron chinense* Schneid (Rutaceae) cortex (EC_50_ = 10.4 ± 2.2 μg/ml, SI = 13.4), *Sophora subprostrata* Chun & T. Chen (Fabaceae) radix (EC_50_ = 27.5 ± 1.1 μg/ml, SI = 11.1), and *Paeonia suffruticosa* Andrews (Paeoniaceae) (EC_50_ = 61.9 ± 6.1 μg/ml, SI = 9.7) were the most active ones against the virus, all of which led to a significant reduction in viral replication after 6 h of exposure following infection. The corresponding EC_90_ values were also determined as 55.6 ± 4.2, 37.9 ± 8.8, 5.8 ± 0.6, 23.4 ± 1.2, and 82.2 ± 8.2 μg/ml for *C. racemosa*, *M. azedarach*, *C. chinensis*, *P. chinense*, and *S. subprostrata*, whereas *P. suffruticosa* had a very low antiviral effect. The data obtained revealed that the target of the extracts was viral RNA synthesis rather than viral entry. By the same research group, 19 more plants were later on screened and *Sophora flavescens* Aiton (Fabaceae) radix, *Sanguisorba officinalis* L. (Rosaceae) radix, *Acanthopanax gracilistylus* W.W. Smith (Araliaceae) cortex, and *Torilis arvensis* (Huds.) Link (Apiaceae) fructus were confirmed to be effective on viral replication in MHV-A59-infected cells [16]. The antiviral action for the active extracts belonging to *A. gracilistylus* cortex and *T. arvensis* fructus was explained to occur through inducing cyclooxygenase-2 (COX-2) expression via the activation of extracellular signal-related kinase (ERK) and p38 or ERK alone.

Essential oils are well-known to have strong antimicrobial activity. In this sense, the essential oils obtained from *Laurus nobilis* L. (Lauraceae), *Juniperus oxycedrus* L. subsp. *oxycedrus* L., *Thuja orientalis* L., and *Cupressus sempervirens* subsp. *pyramidalis* (O. Targ. Tozz.) Nyman from Cupressaceae, *Pistacia palaestina* Boiss. (Anacardiaceae), *Salvia officinalis* L. and *Satureja thymbra* L. from Lamiaceae of Lebanese origin were tested against SARS-CoV (FFM-1 isolate) by determining their CPE during post-infection [17]. The results displayed that the essential oil of *L. nobilis* possessed the strongest activity (IC_50_ = 120 ± 1.2 μg/ml, SI = 4.2), followed by *T. orientalis* (IC_50_ = 130 ± 0.4 μg/ml, SI = 3.8), and *J. oxycedrus* subsp. *oxycedrus* (IC_50_ = 270 ± 1.5 μg/ml, SI = 3.7), where glycyrrhizin as the reference compound had IC_50_ value of 641.0 μg/ml with SI = 1.2. Cellular cytotoxicity of the essential oils tested ranged between 120 to 1000 μg/ml in Vero cells. Phytochemical characterization of the essential oil of *L. nobilis* by gas chromatography-mass spectrometry (GC–MS) led to presence of β-ocimene, 1,8-cineole, α-pinene, and β-pinene as the major compounds.

A Taiwanese team reported strong anti-SARS-CoV (FFM 1 isolate) activity of the tender leaf extracts of *Toona sinensis* Roem (TSL) (syn. *Cedrela sinensis* Juss., Meliaceae) [18]. Their CPE was determined after 72 h of infection and reduction in viral load was measured. A fraction of crude extract of (TSL-1) and the nanoparticulated extract (TSL-nm) of the leaves prepared by boiling and regular techniques were tested separately. TSL-1 (EC_50_ = 30 μg/ml) prepared in regular condition had a greater SI (17) than that of the boiled extract of TSL-1 (> 12) (EC_50_ = 43 μg/ml), where SI (> 13) of TSL-nm (EC_50_ = 37 μg/ml) prepared in regular condition was also higher than that of TSL-nm (SI > 7, EC_50_ = 70 μg/ml). The authors concluded TSL extracts with a promising anti-SARS-CoV activity whose active constituents were not cleared. Zhuang et al. screened the extracts of *Cinnamomum verum* J.S. Presl. (Lauraceae) cortex, *Syzygium aromaticum* (L.) Merrill et L.M. Perry (Myrtaceae) (also known as Caryophylli Flos), *Forsythia suspense* (Thunb.) Vahl. (Oleaceae) fructus, *Scutellaria baicalensis* Georgi (Lamiacea) radix, *Astragalus membranaceous* (Fisch.) Bge. var. *mongholicus* (Bge.) Hsiao (Fabaceae) radix, *Bupleurum chinensis* DC. (Apiaceae) radix, and *Glycyrrhiza uralensis* Fisch. radix against SARS-CoV infection [19]. Among them, *C. verum* (IC_50_ = 30.3 ± 2.6 μg/ml, SI = 6.6) and *S. aromaticum* (IC_50_ = 58.8 ± 5.6 μg/ml, SI = 12.9) showed the highest inhibition in dose-dependent manner. Both extracts were further subjected to chromatographic separation, where the butanol fraction of *C. verum* cortex was revealed as the most active one (IC_50_ = 7.8 ± 0.3 μg/ml, SI = 23.1) against SARS-CoV by the mechanism of prevention of viral entry. Separation of the butanol fraction led to identification of cinnamtannin B1 (IC_50_ = 32.9 ± 2.8 μg/ml, SI = 7.4), procyanidin A2 (IC_50_ = 120.7 ± 13.1 μg/ml, SI = 6.6), and B1 (IC_50_ = 161.1 ± 20.3 μg/ml, SI = 4.1). Besides *trans*-cinnamic acid (IC_50_ = 3.0 ± 0.18 μg/ml, SI = 7.4), 2-phenylethanol (phenethyl alcohol) (IC_50_ = 4.1 ± 0.20 μg/ml, SI = 6.0), and 2-hydroxycinnamic acid (IC_50_ = 0.3 ± 0.01 μg/ml, SI = 8.3) detected to be present in this extract also showed a mild suppressive effect on SARS-CoV infection.

In another screening study, the ethanol extracts of *Taxus celebica* (Warb.) H.L. Li (Taxaceae), *Rheum palmatum* L. (Polygonaceaea), *Sophora flavescens* Aiton (Fabaceae), *Glycyrrhiza uralensis* Fisch. (Fabaceae), *Uvaria microcarpa* Champ. ex Benth. (Annonaceae), *Rubus suavissimus* S. Lee (Rosaceae), *Auricularia auricula* (L.) Underw (Auriculariaceae), Brucea javanica (L.) Merr (Simaroubaceae), male silkworm moths, *Mangifera indica* L. (Anacardiaceae), *Cyrtomium fortunei* (J. Smith) Makino, and *Scutellaria baicalensis* Georgi (Lamiaceae) from China were tested against SARS-CoV 3CL protease (3CL^pro^) [20]. The results showed that *R. palmatum* (RH) had the strongest inhibition and subjected to fractionation through chromatographic separation. Among the fractions, RH10 (IC_50_ = 38.09 ± 1.70 μg/ml), RH11 (IC_50_ = 22.30 ± 1.26 μg/ml), RH12 (IC_50_ = 59.33 ± 6.52 μg/ml), RH121 (IC_50_ = 13.76 ± 0.03 μg/ml), RH122 (IC_50_ = 34.01 ± 5.68 μg/ml), RH124, (IC_50_ = 52.43 ± 4.52 μg/ml), and RH125 (IC_50_ = 20.53 ± 3.20 μg/ml) were promising to inhibit SARS-CoV 3CL^pro^. The main extract had also a very low cytotoxicity up to 20 mg/ml. Antiviral activity of the flowering cherry cultivars including *Prunus yedoensis* ‘Somei-yoshino’, *P. sargentii* ‘Columnaris’, *P. lannesiana* cv. Kawazu-zakura, and *P. cerasus* L. from Rosaceae from Korea was evaluated against porcine epidemic diarrhea virus (PEDV), a kind of coronaviruses, in Vero cells [21]. *P. cerasus* emerged as the most active one with 50% CPE inhibition at 1.95 μg/ml concentration by inhibiting viral replication.

The extracts prepared from the flowers and buds of *Anthemis hyalina* DC. (Asteraceae), the seeds of *Nigella sativa* L. (Ranunculaceae), and the peels of *Citrus sinensis* L. (Rutaceae) from Turkey were tested against MHV-A59 type of betacoronavirus [22]. The maximum non-toxic dose was found to be with 1/50 dilution of the extracts. Exposure of the extracts in virus-infected HeLa-CEACAM1a (HeLa-epithelial carcinoembryonic antigen-related cell adhesion molecule 1a) cells instigated a decrease in virus load, among which *A. hyalina* extract was the most effective. All of the extracts were revealed to modulate transient receptor potential ankyrin 1 (TRPA1), transient receptor potential channel 4 (TRPC4), transient receptor potential cation channel subfamily M (TRPM6), TRPM7, TRPM8, and transient receptor potential cation channel subfamily V (TRPV4) gene expression. Besides, adding of the plant extracts to CoV-infected cells augmented intracellular calcium levels. When extracellular virus release was detected, *A. hyalina* extract was observed to have the highest effect as there was no virus detectable after its exposure.

The ethanol extracts of *Rhodiola rosea* L. (Crassulaceae) roots, *Nigella sativa* L. (Ranunculaceae) seeds, and *Sambucus nigra* L. (Adoxaceae) fruits were tested against infectious bronchitis virus (IBV), known as a pathogenic chicken coronavirus [23]. The extracts were treated for 24 h prior to infection in the cells and their CPE was determined. The results showed that *S. nigra* extract had ability to block infection due to IBV at early stage. Moreover, the virions exposed to *S. nigra* extract were detected to have damaged membrane envelopes. Polyphenols or lectins present in the extract were suggested to be possibly responsible for its antiviral effect via probably interacting with viral proteins such as spike protein, although it still remains unclear. In a recent study, the ethanol extracts prepared from 15 common medicinal plants including *Chamaemelum nobile* (L.) All. and *Echinacea purpurea* (L.) Moench. from Asteraceae, *Satureja montana* L., *Perilla frutescens* L. (Britt.), *Agastache foeniculum* (Pursh) Kunte, *Origanum vulgare* L., *Mentha piperita* L., *Melissa officinalis* L., *Thymus vulgaris* L., *Hyssopus officinalis* L., *Nepeta cataria* L., and *Salvia officinalis* L. from Lamiaceae, *Geranium macrorrhizum* L. (Geraniaceae), *Angelica archangelica* L. (Apiaceae), and *Desmodium canadense* (L.) DC. (Fabaceae) growing in Lithuania were tested against IBV (Vero-adapted Beaudette strain) [24]. When the extracts were treated prior to the infection, the extracts of *C. nobile*, *P. frutescens*, *A. foeniculum*, *O. vulgare*, and *M. piperita* were active, whereas *O. vulgare*, *M. piperita*, *M. officinalis*, *T. vulgaris*, *H. officinalis*, *S. officinalis*, and *D. canadense* were observed to cause inhibitory effect against infection with IBV. *S. montana* extract was the only one active after the infection. Corresponding EC_50_ values were calculated as 0.044, 0.008, 0.004, 0.015, 0.010, 0.076, 0.003, and 0.017 μg for *S. montana* (SI = 17.0), *O. vulgare* (SI = 65.0), *M. piperita* (SI = 67.5), *M. officinalis* (SI = 39.3), *T. vulgaris* (SI = 63.1), *H. officinalis* (SI = 8.4), *S. officinalis* (SI = 36.7), and *D. canadense* (SI = 17.1). The data revealed that the lowest cytotoxicity by 3-(4,5-dimethylthiazol-2-yl)-2,5-diphenyltetrazolium bromide (MTT) assay and the lowest anti-IBV effect were caused by the extracts of *H. officinalis* and *S. montana* among the active extracts. On the other hand, the extracts of *M. piperita*, *D. canadense*, and *T. vulgaris* displayed the highest inhibition on viral replication, and totally blocked viral production at 1 to 0.25 log_10 CC50 (cytotoxic concentration)._

### Natural Molecules from Plants

#### Saponin Derivatives

**Fig. 1:**
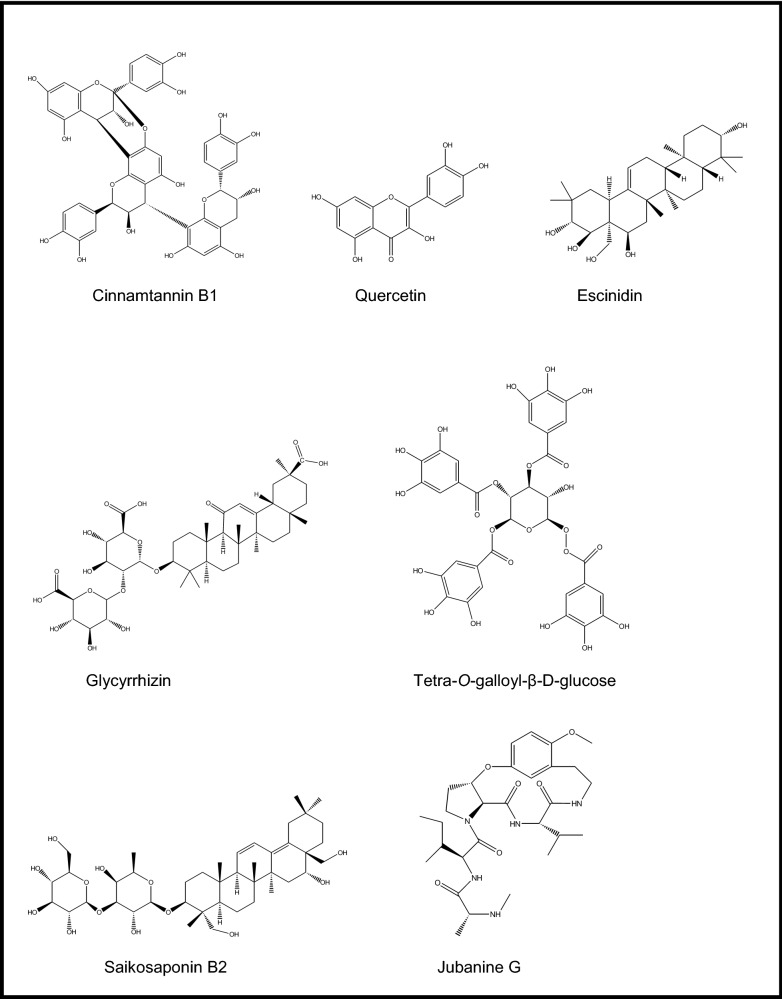

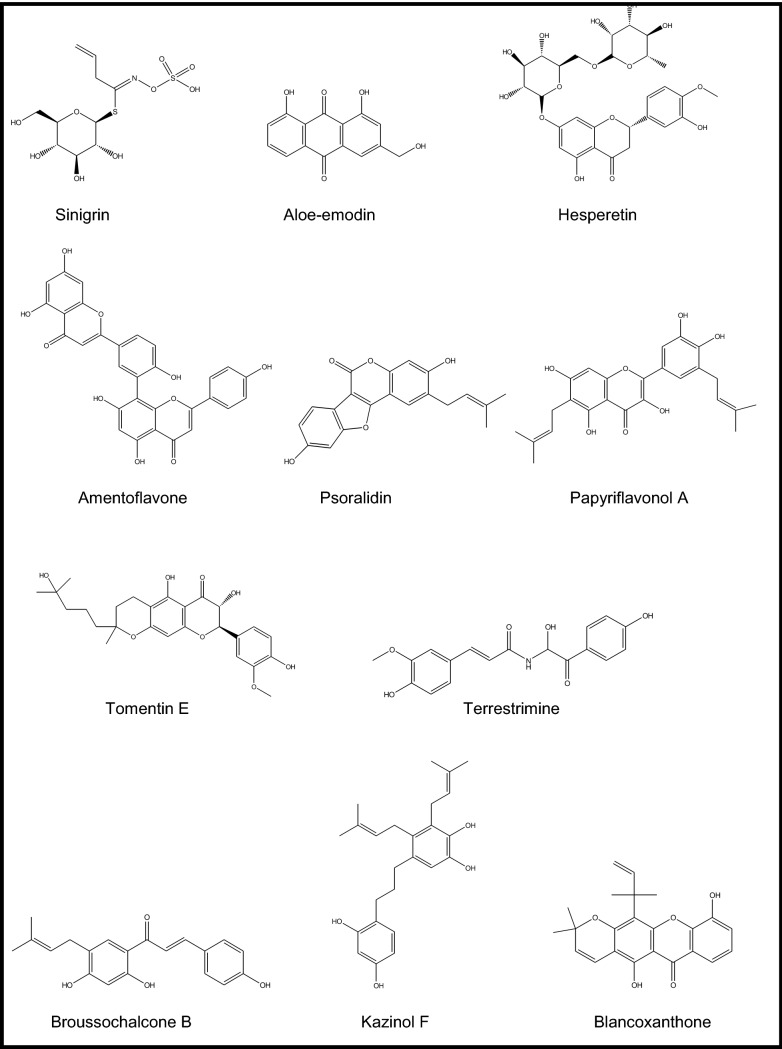

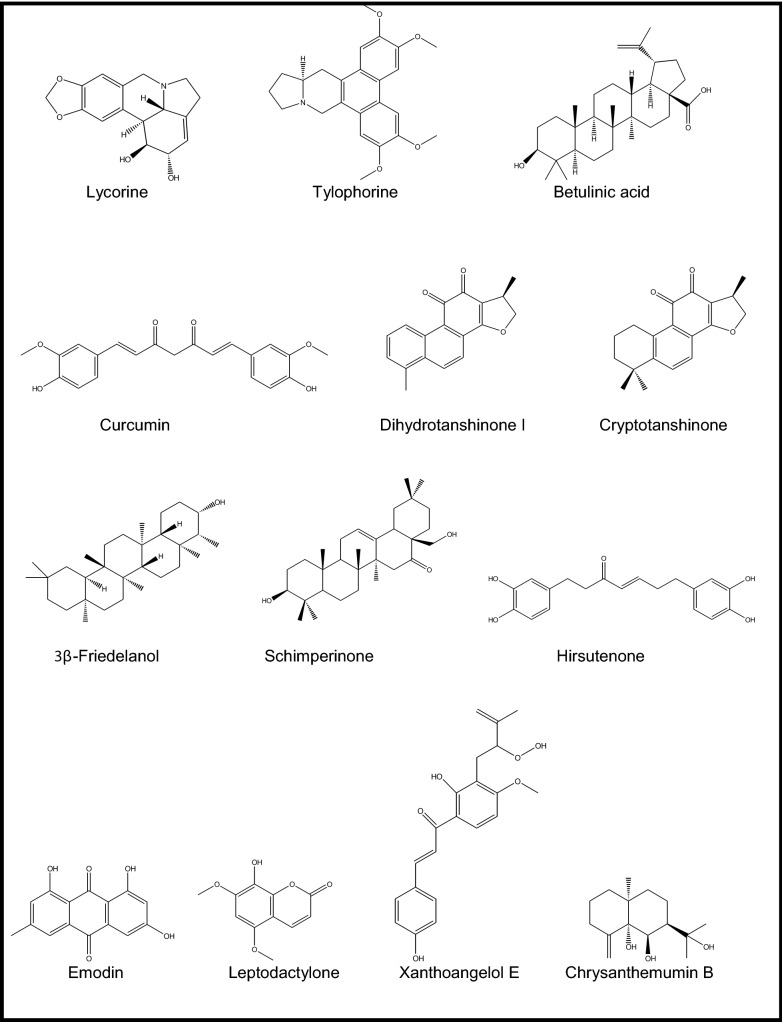
Chemical structures of some promising natural molecules

*Glycyrrhiza* species (licorice) have been reported to have antiviral activity against various viruses [25]. Relevantly, in a study by Cinatl et al. [26], ribavirin, 6-azauridine, pyrazofurin, mycophenolic acid, and glycyrrhizin (Fig. 1), a saponin derivative found in licorice root (*Glycyrrhiza glabra* L., Fabaceae), were tested against two clinical isolates of coronavirus (FFM-1 and FFM-2) obtained from the serums of the SARS-CoV patients at the clinical center of Frankfurt University. Among them, glycyrrhizin, which was previously shown to inhibit HIV-1 and hepatitis C virus, was demonstrated to successfully block replication at early stage, adsorption, and penetration of SARS-type of coronavirus in Vero cells with a SI of 67. It was found to be less active when added during the virus adsorption (EC_50=_ 600 mg/L), whereas it had the highest inhibition when added after the virus adsorption (EC_50=_ 300 mg/L, 364.5 μM). Besides, glycyrrhizin led to less viral antigen expression when the cells were treated with 1000 mg/L of the compound. The possible mechanism of activity by glycyrrhizin was commented as induction of nitric oxide synthase by the authors.

Approximately 10,000 compounds including natural products were investigated for their antiviral potential against SARS-CoV to discover new lead molecules using a cell-based assay in Vero E6 cells via the immunofluorescence (IFA), western blot and flow cytometry analysis techniques [27]. The work also targeted inhibition of SARS-CoV 3C^pro^, which is known to facilitate the proteolytic catalysis of replicase polypeptides into functional proteins with a marked role for viral replication. In the screening, the most active SARS-CoV 3C^pro^ inhibitors were elucidated as escin (a saponin mixture obtained from horse chesnut, *Aesculus hippocastanum* L., Hippocastanaceae) and reserpine (an alkaloid derivative in *Rauwolfia* species) (EC_50_ = 6.0 μM and 3.4 μM, SI = 2.5 and 7.3, respectively). Later on, a similar study against PEDV was conducted on escin (a saponin mixture) obtained from the seeds of *Aesculus turbinata* Blume (Japanese horsechesnut, Hippocastanaceae) by Kim et al. [28]. The chromatographic separation of the escin mixture yielded 10 compounds, 4 of which were new. Screening of the compounds including escinidin, (3β,16α,21β,22α)-16,21,22,28-tetrahydroxyolean-12-en-3-O-β-D-glucopyranosiduronic acid, (3β,16α,21β,22α)-16,21,22,28-tetrahydroxyolean-12-en-3-yl-O-[β-D-glycopyranosyl-(1 4)]-β-D-glucopyranosiduronic acid, (3β,16α,21β,22α)-16,21,22,24,28-pentahydroxy-olean-12-en-3-O-β-D-glucopyranosiduronic acid, (3β,16α,21β,22α)-16,21,22,24,28-pentahydroxyolean-12-en-3-yl-O-[β-D-glycopyranosyl-(1 4)]-β-D-glucopyranosiduronic acid, and aesculuside B did not display cytotoxicity at 20 μM in Vero cells. Considering inhibitory capacity of the compounds on viral replication of PEDV, escinidin was the most potent one at concentrations of 10, 20, and 40 μM, which also significantly inhibited nucleocapsid protein synthesis. Escinidin also prevented RNA expression in both spike and nucleocapsid proteins of the virus. Inhibitory activity of escinidin was further observed on replication of PEDV in a dose-dependent manner at concentrations of 10, 20, and 40 μM using immunochemistry assay. The docking experiments performed with SARS-CoV 3C^pro^ were consistent with the antiviral effect of escinidin, showing clear interactions with the key amino acids such as Glu166, Cys145, and Leu127 in the enzyme.

Saikosaponins A, B2, C, and D, a series of saponin derivatives present in several plant species, *e.g.* the genera *Bupleurum*, *Heteromorpha*, and *Scrophularia*, were tested against SARS-CoV-229E in human fetal lung fibroblasts (MRC-5; ATCC CCL-171) using 2,3-bis[2-methoxy-4-nitro-5-sulfophenyl]-5-[(phenylamino) carbonyl-2H-tetrazolium hydroxide] (XTT), attachment, and penetration assays [29]. No cytotoxic effect was observed with saikosaponins A, B2, C, and D up to concentration of 25 μmol/L, where, similarly, all four saponins exhibited a significant inhibition towards SARS-CoV-229E at this concentration. The most effective one was to be saikosaponin B2 (Fig. 1) causing 100 ± 0.2% of inhibition at 25 μmol/L. EC_50_ values for saikosaponins A, B2, C, and D were determined to be 8.6 ± 0.3 (SI = 26.6), 1.7 ± 0.1 (SI = 221.9), 19.9 ± 0.1 (SI = 19.2), and 13.2 ± 0.3 μmol/L (SI = 13.3), respectively, where actinomycin D (the reference compound) had EC_50_ value of 0.02 ± 0.0 μmol/L (SI = 140). The CC_50_ values (ranging between 121.5–383.3 μmol/L) were better than that of actinomycin D (CC_50_ = 2.8 μmol/L). The further mechanistic studies performed on saikosaponin B2 with the strongest activity confirmed its inhibitory effect on virus-host cell attachment and penetration in dose- and time-dependent pattern affecting early stage of SARS-CoV-229E replication.

#### Flavonoids and Other Polyphenols

Yi et al. targeted SARS-CoV S2 protein, which takes a critical role in entry process of the virus into host cell by developing a two-step screening method combining frontal affinity chromatography-mass spectrometry (FAC/MS) and pseudo-typed virus infection assay [30]. The purpose was to determine if some small molecules can prevent penetration of the virus into host cells (Vero E6 cells) or they can bind to S2 protein in the virus. As the screening materials, small molecules of herbal origin (> 130) were selected from 121 Chinese herbs, which were previously identified to be active against various viruses such as HIV-1, RSV, and hepatitis B virus. For this purpose, a fusion protein (GST-S2) sequenced to Asn733 to Gln1190 as a fragment of S2 protein was produced and its purity and functionality were checked by sodium dodecyl sulfate-poly-acrylamide gel electrophoresis (SDS-PAGE) along with ELISA methods. Binding affinity of the selected small herbal molecules was monitored by FAC/MS. Finally, two molecules, *e.g.* tetra-*O*-galloyl-β-D-glucose (Fig. 1) and luteolin, came out to be the hits from this screening. The further specifity studies pointed out to the fact that these two molecules, particularly tetra-*O*-galloyl-β-D-glucose, were exceedingly specific against SARS-CoV. Their antiviral activity was further tested by MTT assay and compared to those of glycyrrhizin and ribavirin with already known anti-SARS-CoV activity. The findings showed that tetra-*O*-galloyl-β-D-glucose and luteolin inhibited SARS-CoV infection in a dose-dependent manner, having EC_50_ values of 4.5 and 10.6 μM, respectively, where EC_50_ value of glycyrrhizin was > 607.6 μM in the mentioned study. Therefore, tetra-*O*-galloyl-β-D-glucose and luteolin could be considered as promising virus entry inhibitors for SARS-CoV targeting its S2 protein. In the same study, the authors also suggested that quercetin had antiviral activity against HIV-luc/SARS with an EC_50_ of 83.4 μM with a very stumpy toxicity, which could be a good candidate as a natural anti-SARS agent.

The roots of *Isatis indigotica* Fort. (Brassicaceae) growing in Taiwan was investigated against SARS-CoV 3CL^pro^ [31]. The natural molecules including indican (indoxyl-β-d-glucoside), β-sitosterol, sinigrin (Fig. 1), indican, and indirubin, found in the aqueous root extract of the plant along with the extract were subjected to cell-free and cell-based cleavage assays using recombinant SARS-CoV 3CL^pro^. Their cleavage ability was observed through their IC_50_ values. The root extract was active in inhibiting *cis*-cleavage activity of the SARS-CoV 3CL^pro^ (IC_50_ = 191.6 ± 8.2 μg/ml) in cell-based assay, while it was two-fold less effective in cell-free assay. Considering all five compounds from this plant, only sinigrin, β-sitosterol, and indigo were able to inhibit inhibited cleavage function of the 3CL^pro^ in both cell-free and cell-based assays in a dose-dependent style. IC_50_ values of sinigrin, β-sitosterol, and indigo were respectively calculated as 121 μM, 115 μM, and 300 μM in cell-free assays. Their corresponding IC_50_ values were 217 μM, 1210 μM, and 752 μM in cell-based assays. CC_50_ value of the root extract tested in Vero cells was over 5000 μg/ml, while indigo and sinigrin were not also cytotoxic. Among them, sinigrin was found to have a strong correlation between the effects on cell-free and cell-based cleavage of the SARS-CoV 3CL^pro^. Apart from those compounds abovementioned, some other plant phenolics such as aloe-emodin, hesperetin, quercetin, naringenin, daidzein, emodin, and chrysophanol were examined within the same study for their capacity against cleavage of the SARS-CoV 3CL^pro^ in the same manner (Fig. 1). The assay outcomes revealed that only aloe-emodin and hesperetin were able to block cleavage of SARS-CoV 3CL^pro^ dose-dependently. IC_50_ values for hesperetin as the most potent compound among all tested ones were 60.3 μM and 8.3 μM in cell-free and cell-based assays, respectively. Therefore, sinigrin and hesperetin were concluded to be potential lead molecules against SARS-CoV.

Forsythoside A isolated from the fruits of *Forsythia suspensa* Vahl. (Oleaceae) used in traditional Chinese medicine as a major polyphenolic compound was revealed to have antiviral effect on IBV, a kind of coronavirus, obtained from China Institute of Veterinary Drug Control [32]. Anti-IBV activity of the compound on cell viability and proliferation was tested in chicken embryo kidney (CEK) cell cultures. The findings demonstrated that forsythoside A blocked the infection at 0.64 mM in dose-dependent style, which was confirmed by real time-PCR. The leaf extract of *Torreya nucifera* (L.) Siebold et Zuccarini (Taxaceae) from Korea displayed a good level of SARS-CoV 3CL^pro^ inhibitory activity (62% at 100 μg/mL) [33]. Bioactivity-guided isolation of the extract yielded an active flavonoid identified as amentoflavone (Fig. 1) (IC_50_ = 8.3 μM) in non-competitive inhibition manner, followed by luteolin (IC_50_ = 20.2 μM), quercetin (IC_50_ = 23.8 μM), and apigenin (IC_50_ = 280.8 μM). The structure–activity relation study pointed out that methylation of 7-, 4′-, and 4′''-hydroxyl groups present in other biflavonoids (bilobetin, ginkgetin, and sciadopitysin) led to decrease in the activity, while amentoflavon with simple skeleton was found to be more active as supported by molecular docking analysis.

High inhibitory effect of the seed ethanol extract of *Psoralea corylifolia* L. (Fabaceae) from Korea (IC_50_ = 15 μg/ml) against SARS-CoV (papain like) PL^pro^ led to isolation of six active flavonoids, *e.g.* bavachinin (IC_50_ = 38.4 ± 2.4 μM), neobavaisoflavone (IC_50_ = 18.3 ± 1.1 μM), isobavachalcone (IC_50_ = 7.3 ± 0.8 μM), 4′-O-methylbavachalcone (IC_50_ = 10.1 ± 1.2 μM), psoralidin (IC_50_ = 4.2 ± 1.0 μM) (Fig. 1), and corylifol A (IC_50_ = 32.3 ± 3.2 μM) through bioactivity-guided fractionation [34]. However, the characteristic two constituents of this plant known as psoralen and isopsoralen having IC_50_ > 150 μM did not show any effect against SARS-CoV PL^pro^. The flavonoids showed a dose-dependent mode and mixed-type of inhibition.

The fruits of *Paulownia tomentosa* (Thunb.) Steud (Paulowniaceae) from Korea yielded 12 active natural products as SARS-CoV PL^pro^ inhibitors, 5 of which were new geranylated flavonoids, *e.g.* tomentin A (IC_50_ = 6.2 ± 0.04 μM), tomentin B (IC_50_ = 6.1 ± 0.02 μM), tomentin C (IC_50_ = 11.6 ± 0.13 μM), tomentin D (IC_50_ = 12.5 ± 0.22 μM), and tomentin E (IC_50_ = 5.0 ± 0.06 μM) (Fig. 1) in addition to 3′-O-methyldiplacol (IC_50_ = 9.5 ± 0.10 μM), 4′-O-methyldiplacol (IC_50_ = 9.2 ± 0.13 μM), 3′-O-methyldiplacone (IC_50_ = 13.2 ± 0.14 μM), 4′-O-methyldiplacone (IC_50_ = 12.7 ± 0.19 μM), mimulone (IC_50_ = 14.4 ± 0.27 μM), diplacone (IC_50_ = 10.4 ± 0.16 μM), and 6-geranyl-4′,5,7-trihydroxy-3′,5′-dimethoxyflavanone (IC_50_ = 13.9 ± 0.18 μM) [35]. All compounds tested exhibited a mixed-type of inhibition in a dose-dependent manner, which the dihydro-2H-pyran group-containing derivatives were found to be more effective than the rest.

Anti-SARS-CoV PL^pro^ inhibitory activity-guided fractionation of the fruit extract of *Tribulus terrestris* L. (Zygophyllaceae) from Korea yielded six active cinnamic amides recognized as *N*-*trans*-caffeoyltyramine (IC_50_ = 44.4 ± 0.6 μM), *N*-*trans*-coumaroyltyramine (IC_50_ = 38.8 ± 0.4 μM), *N*-*trans*-feruloyltyramine (IC_50_ = 70.1 ± 0.7 μM), terrestriamide (IC_50_ = 21.5 ± 0.5 μM), *N*-*trans*-feruloyloctopamine (IC_50_ = 26.6 ± 0.5 μM), and terrestrimine (a new compound) (IC_50_ = 15.8 ± 0.6 μM) with a mixed type of inhibition in a dose-dependent manner [36].

A number of known polyphenols isolated from the root extract of *Broussonetia papyrifera* (L.) Vent. (Moraceae) growing in Korea were tested against SARS-CoV and MERS-CoV 3CL^pro^ and PL^pro^ [37]. The isolated compounds, *e.g.* broussochalcone A (IC_50_ = 88.1 ± 13.0 μM), broussochalcone B (IC_50_ = 57.8 ± 0.5 μM) (Fig. 1), 4-hydroxyisolonchocarpin (IC_50_ = 202.7 ± 3.9 μM), papyriflavonol A (IC_50_ = 103.6 ± 17.4 μM) (Fig. 1), 3′-(3-methylbut-2-enyl)-3′,4,7-trihydroxyflavane (IC_50_ = 30.2 ± 6.8 μM), kazinol A (IC_50_ = 84.8 ± 10.4 μM), kazinol B (IC_50_ = 233.3 ± 6.7 μM), kazinol F (IC_50_ = 43.3 ± 10.4 μM) (Fig. 1), kazinol J (IC_50_ = 64.2 ± 1.7 μM), broussoflavan A (IC_50_ = 92.4 ± 2.1 μM), isoliquiritigenin (IC_50_ = 61.9 ± 11.0 μM), kaempferol (IC_50_ = 116.3 ± 7.1 μM), quercetin (IC_50_ = 52.7 ± 4.1 μM), and quercetin-β-galactoside (IC_50_ = 128.8 ± 4.5 μM) were reported to be notable inhibitors of PL^pro^ in non-competitive type of inhibition mode and dose-dependent manner against SARS-CoV 3CL^pro^. The same compounds had IC_50_ values ranging between 7.6 ± 0.4 μM and 136.9 ± 4.7 μM in deubiquitination assay for PL^pro^, where the most active one was papyriflavonol A. In deISGylation assay to measure PL^pro^ inhibitory effect, IC_50_ values of the compounds varied between 8.5 ± 1.2 μM and 71.7 ± 7.4 μM, being again papyriflavonol A as the most active inhibitor. Considering inhibition of MERS-CoV proteases, the compounds possessed IC_50_ values between 27.9 ± 1.2 μM and 193.7 ± 15.6 μM for 3CL^pro^ and 42.1 ± 5.0 μM and 206.6 ± 1.7 μM for PL^pro^. According to these results, broussochalcone B was the most potent against MERS-CoV 3CL^pro^, while PL^pro^ of MERS-CoV was most effectively inhibited by kazinol F as a biphenylpropanoid derivative. It was also suggested that the positions of the two prenyl groups located on the compounds caused a significant influence on SARS-CoV PL^pro^ inhibition, which indicated existence of hydrophobic interactions with the enzyme. On the other hand, the flavan skeleton caused a decrease in inhibitory potential of these compounds against SARS-CoV PL^pro^.

On the other hand, phytochemical studies performed on the roots of *Calophyllum blancoi* Planch. & Triana (Calopyllaceae) of Taiwanese origin with the traditional use of antimicrobial properties led to isolation of several pyranoxanthone derivatives [38]. Among them, blancoxanthone (Fig. 1) as well as pyranojacareubin was revealed to possess a remarkable anti-coronavirus activity against human coronavirus 229E (HCoV 229E) with EC_50_ values of 3 and 15 μg/ml, respectively, in human lung fibroblast (MRC-5) cell line using (XTT) assay.

13 molecules present in 230 Chinese herbs used traditionally against respiratory diseases were investigated for their probable effect against SARS-CoV-2 using in silico techniques [39]. The compounds (betulinic acid, coumaroyltyramine, cryptotanshinone, desmethoxyreserpine, dihomo-c-linolenic acid, dihydrotanshinone I, kaempferol, moupinamide, *n*-*cis*-feruloyltyramine, quercetin, sugiol, and tanshinone IIa) selected through a multiple step selection process using a network pharmacological analysis from several databases were applied in silico against SARS-CoV PL^pro^ and 3CL^pro^ inhibition, viral replication, and viral entry as the targets and proposed to be hopeful for the treatment of novel coronavirus causing COVID-19. In a similar work published very recently, Ul Qamar et al. screened a collection of 32,297 potential antiviral phytochemicals from traditional Chinese medicine using SARS-CoV-2 CL^pro^ [40]. Its sequence was constructed its 3D homology model based on the similarity to that of SARS-CoV-1. The docking analyses pointed out to discovery of 9 hit natural compounds, proposed as myricitrin from *Myrica cerifera* L. (Myricaceae), methyl rosmarinate from *Hyptis atrorubens* Poit (Lamiaceae), 5,7,3′,4′-tetrahydroxy-2′-(3,3-dimethylallyl) isoflavone from *Psorothamnus arborescens* (Torr. ex A.Gray) Barneby (Fabaceae), 3,5,7,3′,4′,5′-hexahydroxy flavanone-3-O-β-D-glucopyranoside from *Phaseolus vulgaris* L. (Fabaceae), (2S)-eriodictyol 7-O-(6′'-O-galloyl)-β-D-glucopyranoside from *Phyllanthus emblica* L. (Phyllanthaceae), calceolarioside B from *Fraxinus sieboldiana* Blume (Oleaceae), myricetin 3-O-β-D-glucopyranoside from *Camellia sinensis* (L.) Kuntze (Theaceae), licoleafol from *Glycyrrhiza uralensis* Fisch & DC. (Fabaceae), and amaranthin from *Amaranthus tricolor* L. (Amaranthaceae).

#### Alkaloids

Over 200 medicinal plants of Chinese origin were screened against SARS-CoV (strain BJ001) using 3-(4,5-dimethylthiazol-2-yl)-5-(3-carboxymethoxyphenyl)-2-(4-sulfophenyl)-2H-tetrazolium inner salt (MTS) assay in Vero E6 cells [41]. Out of them, *Lycoris radiata* (L.’Her) Herb. (Amaryllidaceae), *Artemisia annua* L. (Asteraceae), *Pyrrosia lingua* (Thunb.) Farw. (Polypodiaceae), and *Lindera aggregata* (Sims) Kosterm. (Lauraceae) (EC_50_ = 2.1, 39.2, 40.5, and 80.6 IU/ml, respectively) were found to inhibit virus-induced CPE markedly through replication and infection of the virus in dose-dependent pattern as compared to that of interpheron-α (EC_50_ = 587.2 IU/ml, SI = > 170). Being the most potent one, *L. radiata* was shown to have SI of 422, followed by *P. lingua* (SI = 59) and *A. annua* (SI = 27). Then, the alkaloid-rich fraction of *L. radiata* (EC_50_ = 1 IU/ml, SI = 94) was further subjected to chromatographic isolation procedure, which led to identification of lycorine (Fig. 1), as an active alkaloid against SARS-CoV with EC_50_ value of 15.7 IU/ml (SI = 954). Thus, lycorine was suggested as an encouraging natural antiviral agent against this virus by the authors.

A series of phenanthroindolizidines and phenanthroquinolizidine derivatives (tylophorine, tylocrebrine, and tylophorinine, the characteristic compounds of the genera *Cynanchum*, *Pergularia*, and *Tylophora* as well as some other genera of the Asclepiadaceae family) of natural and synthetic origins, 18 in total, were evaluated as possible inhibitors of transmissible gastroenteritis virus (TGEV) from porcine, a type of enteropathogenic coronaviruses using IFA and determination of CPE [42]. EC_50_ values of the effective compounds varied between 8 and 1468 nM. The most potent ones were tylophorine (EC_50_ = 58 ± 4 nM, SI = > 1715) (Fig. 1) and 7-methoxycryptopleurine (EC_50_ = 20 ± 1 nM, SI = 2232), which displayed TGEV replication activity via preventing the TGEV-induced apoptosis and subsequent cytopathic effect in swine testicular epithelial cells with high oral bioavailability. Necessity of a hydroxyl group at C14 or at the phenanthrene moiety at C3 was shown for the mentioned effect for tylophorine derivatives, while an oxygen atom introduced to the reactive nitrogen to form an *N*-oxide caused a decrease in antiviral activity.

Jubanines F-J identified as cyclopeptide alkaloids and three known compounds, nummularine B, daechuine-S3, and mucronine K isolated from the root extract of *Ziziphus jujuba* Mill. (Rhamnaceae) from Korea were tested against porcine epidemic diarrhea virus (PEDV) in Vero cells [43]. Jubanine G, H, and nummularine B were identified as the most potent alkaloids with the EC_50_ and SI values of 13.41 ± 1.13 μM, SI = > 30.04 ± 2.74), 4.49 ± 0.67 μM (SI = 47.11 ± 0.49), and 6.17 ± 0.50 Μm (SI = 26.75 ± 0.54), respectively, as compared to azauridine as the reference compound (EC_50_ = 5.58 ± 0.53 μM, SI = 7.98 ± 0.37). Moreover, lower cytotoxicity was observed with jubanine G (Fig. 1), H, and nummularine B (CC_50_= > 400 μM, 211.26 ± 29.64 μM, and 165.30 ± 16.49 μM, respectively), when compared to that of the reference (44.47 ± 6.11 μM) using MTT assay. Rest of the isolated compounds determined as jubanine F, I, J, daechuine-S3, and mucronine K did not display antiviral activity against PEDV.

#### Terpenes

Wen et al. conducted a similar survey on 221 natural products from plants covering mostly terpene- and lignin-derivatives to measure their antiviral potential against SARS-CoV utilizing a Vero E6 cell-based CPE assay [44]. Among them, promising 20 compounds [ferruginol, dehydroabieta-7-one, sugiol, 8β-hydroxyabieta-9(11),13-dien-12-one, 6,7-dehydroroyleanone, pinusolidic acid, α-cadinol, hinokinin, savinin, 3β,12-diacetoxyabieta-6,8,11,13- tetraene, cedrane-3β,12-diol, betulonic acid, betulinic acid, cryptojaponol, 7β-hydroxy-deoxycryptojaponol, 4,4′-*O*-benzoylisolariciresinol, forskolin, curcumin, honokiol, and magnolol] were further evaluated for their inhibitory activity against of SARS-CoV 3CL^pro^ through molecular modelling. The analysis showed that the active compounds having anti-SARS-CoV activity included abietane-type [ferruginol, dehydroabieta-7-one, sugiol, 8β-hydroxyabieta-9(11),13-dien-12-one, 6,7-dehydroroyleanone, and 3β,12-diacetoxyabieta-6,8,11,13-tetraene] and labdane-type diterpenes (pinusolidic acid and forskolin), sesquiterpenes (cedrane-3β,12-diol and α-cadinol), lupane-type triterpenes (betulinic acid), lignoids (hinokinin, savinin, 4,4′-*O*-benzoylisolariciresinol, honokiol, and magnolol), and curcumin. Among them, only betulinic acid (a lupane-type of triterpene, Ki = 8.2 ± 0.7 μM) (Fig. 1) and savinin (a lignan derivative, Ki = 9.1 ± 2.4 μM) had the capacity to block SARS-CoV 3CL^pro^ on competitive basis.

SARS-CoV 3CL^pro^ inhibitory potential of four quinone-methide triterpenoid derivatives elucidated as celastrol (IC_50_ = 10.3 ± 0.2 μM), pristimerin (IC_50_ = 5.5 ± 0.7 μM), tingenone (IC_50_ = 9.9 ± 0.1 μM), and iguesterin (IC_50_ = 2.6 ± 0.3 μM) isolated from *Tripterygium regeli* Hook F (Celastraceae) of Korean origin (known as thunder god vine), also native to China, Japan, and Taiwan, as well as the synthesized one, dihydrocelastrol (IC_50_ = 21.9 ± 1.9 μM), was reported in comparison to that of curcumin as the reference compound (IC_50_ = 23.5 ± 3.7 μM) [33]. Their inhibitory activity on SARS-CoV 3CL^pro^ was observed to occur in a competitive and dose-dependent mode. The structure–activity relationship indicated that quinone-methide moiety in these compounds is essential for inhibition of SARS-CoV 3CL^pro^ supported by molecular docking experiments.

Antiviral activity of tanshinones, the major diterpenes found in *Salvia miltiorrhiza* Bunge (Lamiaceae) and salvionolic acid, was investigated through inhibition assays targeting SARS-CoV 3CL^pro^ and PL^pro^ [45]. Except cryptotanshinone (IC_50_ = 226.7 ± 6.2 μM), the rest of the compounds including tanshinone IIA (IC_50_ = 89.1 ± 5.2 μM), tanshinone IIB (IC_50_ = 24.8 ± 0.8 μM), methyl tanshinonate (IC_50_ = 21.1 ± 0.8 μM), tanshinone I (IC_50_ = 38.7 ± 8.2 μM), dihydrotanshinone I (IC_50_ = 14.4 ± 0.7 μM), and rosmariquinone (IC_50_ = 21.1 ± 0.8 μM) exhibited a dose-dependent inhibitory effect on 3CL^pro^ in non-competitive pattern. Taking dihydrotanshinone I into consideration, the presence of naphthalene in the diterpene quinolone backbone of the compound as well as hydroxymethyl group and methyl ester moiety on the D-ring derivative was shown to have an important role in its inhibitory activity, whereas dihydrofuran moiety on the A-ring to this group decreased the inhibitory activity like in tanshinone I. On the other hand, all tested tanshinones appeared to possess higher inhibitory activity against PL^pro^. Among them, cryptotanshinone (IC_50_ = 0.8 ± 0.2 μM) (Fig. 1) was revealed to be the strongest inhibitor, followed by tanshinone IIA (IC_50_ = 1.6 ± 0.5 μM) and dihydrotanshinone I (IC_50_ = 4.9 ± 1.2 μM). Considering the structure–activity of them against PL^pro^, dimethyl tetrahydronaphthalen structure was stated to enhance the inhibition. The kinetic studies indicated that most of the active compounds had slow-binding inhibition mechanism.

The isolation study on the leaf ethanol extract of *Euphorbia neriifolia* L. (Euphorbiaceae) from Taiwan afforded 22 triterpenoids and one flavonoid glycoside in total [46]. Among them, the strongest inhibitor against human coronavirus (229E strain) was identified as 3β-friedelanol (Fig. 1). Friedelane skeleton in the compound was the key factor in potent inhibition for the lead molecules to develop future anti-coronavirus agents.

Against PEDV, antiviral potential of 15 oleanane triterpenes, 9 of which were identified as new for nature isolated from the flower ethanol extract of *Camellia japonica* ‘Alba Plena’ (Theaceae) from Korea, was investigated [47]. Among them, schimperinone (EC_50_ = 0.28 ± 0.09 μM, SI = 44.54 ± 8.34) (Fig. 1), 3β-hydroxy-28-noroleana-12,17-dien-16-one (EC_50_ = 0.28 ± 0.11 μM, SI = 32.72 ± 6.22), 3β-hydroxy-28-norolean-12,17-dien-16-one 3-O-6′-methoxy-α-D-glucuronopyranoside (EC_50_ = 0.93 ± 0.22 μM, SI = 14.74 ± 1.62), and 3β,16α-dihydroxyolean-12-en-28-al 3-O-β-D-glucuronopyranoside (EC_50_ = 0.34 ± 0.01 μM, SI = 6.68 ± 0.14) were demonstrated to have the strongest inhibition on virus as compared to that of azauridin (EC_50_ = 3.37 ± 0.71 μM, SI = 14.30 ± 1.24). Besides 3β-hydroxy-28-noroleana-12,17-dien-16-one was shown to inhibit viral replication potently in time-course study and found to inhibit PEDV RNA expression dose-dependently by encoding nucleocapsid, spike, and membrane protein, at 2.0, 1.0, 0.5, and 0.25 μM concentrations. The active four compounds were further tested on their probable inhibition on PEDV nucleocapsid and spike protein synthesis during replication. Then 3β-hydroxy-28-norolean-12,17-dien-16-one 3-O-6′-methoxy-α-D-glucuronopyranoside exerted a noteworthy inhibition at concentrations of 8.0, 4.0, 2.0, and 1.0 μM in this assay, which was better than that of azauridin. On the other hand, schimperinone was shown to block PEDV replication in a dose-dependent pattern at concentrations of 4.0, 2.0, and 1.0 μM.

An extensive isolation work on the flower extract of *Chrysanthemum indicum* L. (Asteraceae) afforded 25 sesquiterpenoids from different chemical groups (eudesmane, iphionane, germacrane, and guaiane), where 10 of them (named as chrysanthemumins A − J) were identified as new for nature and 15 were already known analogues [48]. Antiviral effect of the sesquiterpenoids was evaluated against PEDV in Vero cells by comparing to azauridine as the reference compound. The data showed that chrysanthemumins A−E as well as 6,8-cycloeudesm-4(15)-en-1-ol, 11-hydroxy-1-oxo-4α,5α,7β,10β-eremophilane, chrysanthediol A, 1β-hydroxy-4(15),5E,10(14)-germacratriene, ligucyperonol, and eudesm-4(15)-ene-1β,6α-diol were found to own a protective effect against viral infection to some degree. Additionally, chrysanthemumin B (Fig. 1), 6,8-cycloeudesm-4(15)-en-1-ol, and 1β-hydroxy-4(15),5E,10(14)-germacratriene were able to inhibit viral replication at concentrations varying 20–90 mM in dose-dependent style. The mentioned three compounds were further tested on the proteins necessary for PEDV replication. Among them, chrysanthemumin B and 1β-hydroxy-4(15),5E,10(14)-germacratriene blocked expression of nucleocapsid and spike protein synthesis at concentrations of 80, 40, 20, and 10 μM in dose-dependent manner.

#### Anthraquinones

312 Chinese medicinal plants growing in Taiwan were screened to find a hit against SARS-CoV, targeting its spike (S) protein, a type I membrane-bound protein vital for viral entry into host cell through binding to cellular receptors [49]. Out of these Polygonaceae family plants, the root tubers of *Rheum officinale* Baill. along with the root tubers and the vines of *Polygonum multiflorum* Thunb., came out as the most effective extracts (IC_50_ values ranging between 1–10 μg/ml) in biotinylated ELISA, IFA in Vero E6 cells, and MTT assays. Afterward, these three active plants were found to contain emodin (1,3,8-trihydroxy-6-methylanthraquinone) (Fig. 1) and rhein (1,8-dihydroxy-3-carboxyl-9,10-anthraquinone) as two anthraquinone derivatives as well as chrysin (5,7-dihydroxyflavone) in large amounts in common. Emodin caused a decrease in cell-associated fluorescence indicating that it was able to block S protein. It was also able to prevent the S protein-pseudo-typed retrovirus infectivity dose-dependently with 94.12 ± 5.90% at 50 μM. The structure–activity indicated that the side chain in anthraquinone, rather than the anthraquinone skeleton is essential for inhibiting S protein. In another study [50], emodin was shown to have an antiviral effect against feline coronavirus with CC_50_ value of 67.41 μM, however, a strong cytotoxicity was observed.

#### Coumarins and Chalcones

Leptodactylone (Fig. 1), a coumarin derivative isolated from *Boenninghausenia sessilicarpa* H.Lev. (Rutaceae) from China was reported to have antiviral activity against SARS-CoV with ratio of 60% at 100 microg/ml in cell-based assay [51].

Park et al. tested 9 alkylated chalcones and 4 coumarins from the leaf ethanol extract of *Angelica keiskei* Ito (Apiaceae) of Korean origin targeting the SARS-CoV 3CL^pro^ and PL^pro^ by cell-free (CF) and cell-based (CB) cleavage assays [52]. Only the alkylated chalcones elucidated as isobavachalcone (IC_50_ = 39.4 ± 5.2 μM for CF, IC_50_ = 11.9 ± 2.8 μM for CB, SI = 1.3), xanthoangelol (IC_50_ = 81.4 ± 8.5 μM for CF, IC_50_ = 50.8 ± 3.0 μM for CB, SI = 0.4),), 4-hydroxyderricin (IC_50_ = 38.4 ± 3.9 μM for CF, IC_50_ = 5.8 ± 0.6 μM for CB, SI = 3.5), xanthoangelol F (IC_50_ = 34.1 ± 4.8 μM for CF, IC_50_ = 32.6 ± 2.2 μM for CB, SI = 0.6), xanthoangelol D (IC_50_ = 26.6 ± 5.2 μM for CF, IC_50_ = 9.3 ± 1.2 μM for CB, SI = 6.7), xanthoangelol E (IC_50_ = 11.4 ± 1.4 μM for CF, IC_50_ = 7.1 ± 0.8 μM for CB, SI = 9.2) (Fig. 1), xanthoangelol B (IC_50_ = 22.2 ± 6.5 μM for CF, IC_50_ = 8.6 ± 2.6 μM for CB, SI = 4.2), xanthoangelol G (IC_50_ = 129.8 ± 10.3 μM for CF, not tested for CB, SI = not tested), and xanthokeistal A (IC_50_ = 44.1 ± 1.3 μM for CF, IC_50_ = 9.8 ± 2.3 μM for CB, SI = 6.4) displayed a dose-dependent and competitive inhibition towards SARS-CoV 3CL^pro^. Nevertheless, the isolated coumarins (*e.g.* psoralen, bergapten, xanthotoxin, and isopimpinellin) possessed a very low inhibition varying between 10 and 45% at 200 μM. As shown by the data, xanthoangelol E was the most potent against SARS-CoV 3CL^pro^, pointing out to the important role of location of the perhydroxyl (–OOH) group considering high activity of this compound. The compounds exhibited an inhibitory profile against SARS-CoV PL^pro^ in non-competitive mode (except isobavachalcone with mixed-type inhibition). Their IC_50_ and SI values were determined as follows; isobavachalcone (IC_50_ = 13.9 ± 0.9 μM), xanthoangelol (IC_50_ = 11.7 ± 3.2 μM), 4-hydroxyderricin (IC_50_ = 13.9 ± 0.9 μM), xanthoangelol F (IC_50_ = 5.6 ± 0.5 μM), xanthoangelol D (IC_50_ = 19.3 ± 1.8 μM), xanthoangelol E (IC_50_ = 1.2 ± 0.4 μM), xanthoangelol B (IC_50_ = 11.7 ± 0.3 μM), xanthoangelol G (IC_50_ = 46.4 ± 7.8 μM), and xanthokeistal A (IC_50_ = 21.1 ± 5.6 μM). The molecular docking studies also confirmed the enzymatic inhibition assay results.

#### Diarylheptanoids

Park et al. studied inhibitory potential of 9 diarylheptanoid derivatives (platyphyllenone, hirsutenone, platyphyllone, platyphyllonol-5-xylopyranoside, hirsutanonol, oregonin, rubranol, rubranoside B, and rubranoside A) isolated from *Alnus japonica* Steud (Betulaceae) of Korean origin in the same manner against both SARS-CoV 3CL^pro^ and PL^pro^ using a continuous fluorometric assay [53]. Firstly, the stem bark ethanol extract of the plant was identified with a marked PL^pro^ inhibition, which led to isolation of the aforementioned diarylheptanoids containing basically 1,7-diphenylheptane skeleton. Among them, hirsutenone (IC_50_ = 3.0 ± 1.1 μM), hirsutanonol (IC_50_ = 24.1 ± 2.0 μM), oregonin (IC_50_ = 44.5 ± 5.3 μM), rubranol (IC_50_ = 35.2 ± 1.7 μM), rubranoside B (IC_50_ = 7.2 ± 2.2 μM), and rubranoside A (IC_50_ = 14.4 ± 3.0 μM) were found to be the promising inhibitors of SARS-CoV PL^pro^ in deubiquitination activity assay in comparison to that of curcumin (IC_50_ = 5.7 μM) as the reference compound. α,β-Unsaturated carbonyl group with a catechol moiety was correlated with the higher inhibition in these molecules, whereas monohydroxyl substitution led to diminish of the inhibitory effect. Within the same work, the glycoside derivatives of these diarylheptanoids were also tested and found to exhibit poorer inhibition than the diarylheptanoids. When they were tested against recombinant SARS-CoV 3CL^pro^, the findings showed that the diarylheptanoids displayed a noteworthy selectivity towards the coronaviral proteases.

#### Lectins and Polysaccharides

Lectins (also known as plant agglutinins), as another plant metabolite group of protein-type, are known to possess antiviral effect depending on their sugar moiety. In this regard, a collection of 33 specific plant lectins containing mannose, *N*-acetyl glucosamine, glucose, galactose, and *N*-acetyl galactosamine were investigated against SARS-CoV Frankfurt 1 strain (Germany) in Vero E6 cells [54]. Out of them, 15 lectins exhibited anti-SARS-CoV effect to some extent. The mannose-specific plant lectin isolated from leek (APA) was revealed to have the strongest effect (EC_50_ = 0.45 ± 0.08 μg/ml, SI = > 222). APA was followed by *N*-acetyl glucosamine-specific lectins isolated from the stinging nettle (*Urtica dioica* L., Urticaceae) (UDA, EC_50_ = 1.3 ± 0.1 μg/ml, SI = > 77) and from the tobacco plant (*Nicotiana tabacum* L., Solanaceae) (Nictaba, EC_50_ = 1.7 ± 0.3 μg/ml, SI = > 59). Nevertheless, their efficacy was less against SARS-CoV as compared to HIV and feline infectious peritonitis virus (FIPV). It was commented to be related to their low binding capacity on coronavirus envelope glycans as SARS-CoV spike protein contains 12 N-glycosylation sites, four of which were so far identified. In the same study, HHA (EC_50_ = 3.2 ± 2. 8 μg/ml, SI = > 100), the mannose-specific lectin from *Hippeastrum* hybrid (Amaryllidaceae), was selected for further inspection against both SARS-CoV and FIPV. The time-of-addition assay with HHA indicated that the lectin was able to decrease intracellular viral RNA load, when it was added at the time of infection. This result suggested that the plant lectins interfere with the viral replication cycle at early stage affecting its attachment to the specific ACE-2 receptors on the cell surface. In a similar study by Hsieh et al. (2010) [50], *Galanthus nivalis* L. (Amaryllidaceae) agglutinin (GNA) (IC_50_ = 0.0088 nM) via binding to the two glycosylated envelope glycoproteins (*e.g.*, the spike and membrane proteins) was reported to inhibit FIPV. Furthermore, synergistic interaction of GNA with combined use of nelfinavir was observed to inhibit viral infection, where no cytotoxicity was found for this combination.

A polysaccharide isolated from the exocarps of *Ginkgo biloba* L. (Ginkgoceae) from Korea was tested against PEDV [55]. It was found to be the most effective when treated 1 h with PEDV at concentration of 5 μg/ml, which prevented PEDV attachment and entry of the virus to Vero cells in dose-, time-, and temperature-dependent conditions. It was also microscopically proved by morphological changes (IC_50_ = 1.7 μg/ml). On the other hand, the polysaccharide was not toxic to Vero cells (CC_50_ = > 100 μg/mL). Various *Astragalus* species are known to have immunomodulatory and antiviral effects. *Astragalus* polysaccharides (APS) obtained from *Astragalus mongholicus* Bunge (Fabaceae) were tested against IBV (strain M41) in CEK cells [56]. APS was able to kill 47.6% of cells at concentration of 30 μg/ml, where N protein expression observed abundantly in viral proteins as a marker for viral protein production was dose-dependently diminished, when IBV-infected CEK cells were exposed to APS. APS also showed an inhibitory action in IBV-infected CEK cells via lessening the expression of pro-inflammatory cytokines such as interleukin (IL)-1B, IL-6, IL-8, and tumor necrosis factor-α (TNF-α). Relevantly, APS was reported to be a beneficial adjuvant for IBV vaccine by the same research group [57]. Relevantly, ginseng stem-leaf saponins in combination with selenium were reported to improve both IBV- and Newcastle disease virus (NDV)-specific antibody responses by enhancing lymphocyte proliferation and production of interferon (IFN)-γ and IL-4 in chickens, which might be considered as possible adjuvants for the vaccines against the aforementioned viruses [58].

Among plant knottins, alstotides (As1-As4) having 30 amino acid residues were obtained from the leaf aqueous extract of *Alstonia scholaris* (L.) R. Br. (Apocynaceae) and sequenced de novo by tandem MS/MS and clarified by either gene cloning (As1, As2, and As4) or amino acid analysis [59]. They were tested for their probable antiviral effect against IBV, a highly infectious kind of alphacoronaviruses, at early stage. The inhibition by these compounds was shown in Vero cells using combination of time of drug addition, transfection, in vitro pulldown, co-immunoprecipitation, and IFA assays. Among them, As1 was found to act during an early stage of the viral infection, when exposed to infected cells at different time points during pre-incubation (attachment) at 4 °C and the infection (entry) at 37 °C. Furthermore, As1 interacted in vitro with IBV membrane (M) and spike (S) proteins but not nucleocapsid (N) protein. As1 had intracellular association with M protein in infected Vero cells. Since peptides are generally known to have low capability to pass cell membranes, As1, which was found to be not cytotoxic concentrations up to 100 μM, was subjected to cell permeability assay, which indicated that As1 was able to transverse the cell membrane entry into the cells.

## Conclusion

Natural products have been always attractive to scientists for drug research to develop novel candidates. COVID-19 caused by the novel coronavirus (SARS-CoV-2) is a deadly infectious disease against which no specific drug or vaccine is available, yet. Unceasing efforts through experimental and clinical studies are being made to struggle the disease, although several antiviral drugs used against SARS-CoV are currently practiced to treat the patients. Our present review emphasized that the natural products already reported with inhibitory activity towards various types of coronaviruses could be a model for new drug candidates to inhibit SARS-CoV-2. Most of the active natural compounds have been belong to polyphenols and flavonoids (quercetin, luteolin, hesperetin, amentoflavone, tetra-O-gallyl-β-D-glucose, sinigrin, forsythoside A, psoralidin, tomentin B, terrestrimine, broussochalcone, papyriflavonol A). In addition, some alkaloids (lycorine, tylophorine, 7-methoxycryptopleurine, jubanine H, nummularine B), anthraquinones (aloe-emodin, emodin), saponins (glycryrrhizin, escinidin, saikosaponin B2), terpenes (curcumin, betulinic acid, savinin, iguesterin, dihydrotanshinone I, cryptotanshinone, 3β-friedelanol, chrysanthemumin B), coumarins (leptodactylone, xanthoangelol E), diarylheptanoids (hirsutenone), and lectins (APA, UDA, HHA, alstotide 1) seem to be quite promising against SARS-CoV. Most recent in silico studies also revealed that such molecules like myricitrin, methyl rosmarinate, 5,7,3′,4′-tetrahydroxy-2′-(3,3-dimethylallyl) isoflavone, 3,5,7,3′,4′,5′-hexahydroxy flavanone-3-O-β-D-glucopyranoside, (2S)-eriodictyol 7-O-(6′′-O-galloyl)-β-D-glucopyranoside, calceolarioside B, myricetin 3-O-β-D-glucopyranoside, licoleafol, and amaranthin could be potential leads to develop novel anti-SARS-CoV-2 drugs. In conclusion, the natural molecules could give a hopeful way to further COVID-19 drug research.

## References

[1] Weiss SR, Navas-Martin S (2005). Microbiol. Mol. Biol. Rev..

[2] Groneberg D, Hilgenfeld R, Zabel P (2005). Respir. Res..

[3] Ksiazek TG, Erdman D, Goldsmith CS, Zaki SR, Peret T, Emery S, Tong S, Urbani C, Comer JA, Lim W, Rollin PE, Dowell SF, Ling AE, Humphrey CD, Shieh WJ, Guarner J, Paddock CD, Rota P, Fields B, Derisi J, Yang JY, Cox N, Hughes JM, LeDuc JW, Bellini WJ, Anderson LJ, Engl N (2003). J. Med..

[4] Andersen KG, Rambaut A, Lipkin WI, Holmes EC, Garry RF (2020). Nat. Med..

[5] Zhang T, Wu Q, Zhang Z (2020). Curr. Biol..

[6] Tort FL, Castells M, Cristina J (2020). Virus Res..

[7] Lu R, Zhao X, Li J, Niu P, Yang B, Wu H (2020). Lancet.

[8] Ceccarelli M, Berretta M, Venanzi Rullo E, Nunnari G, Cacopardo B (2020). Eur. Rev. Med. Pharmacol. Sci..

[9] Devaux CA, Rolain JM, Colson P, Raoult D (2020). Int. J. Antimicrob. Agents.

[10] Costanzo M, De Giglio MAR, Roviello GN (2020). Curr. Med. Chem..

[11] Stebbing J, Phelan A, Griffin I, Tucker C, Oechsle O, Smith D, Richardson P (2020). Lancet Infect. Dis..

[12] Lu H (2020). Biosci. Trends.

[13] McCutcheon AR, Roberts TE, Gibbons E, Ellis SM, Babiuk LA, Hancock RE, Towers GH (1995). J. Ethnopharmacol..

[14] Kotwal GJ, Kaczmarek JN, Leivers S, Ghebremariam YT, Kulkarni AP, Bauer G, De Beer C, Preiser W, Mohamed AR (2005). Ann. NY Acad. Sci..

[15] Kim HY, Shin HS, Park H, Kim YC, Yun YG, Park S, Shin HJ, Kim K (2008). J. Clin. Virol..

[16] Kim HY, Eo EY, Park H, Kim YC, Park S, Shin HJ, Kim K (2010). Antiviral Ther..

[17] Loizzo MR, Saab AM, Tundis R, Statti GA, Menichini F, Lampronti I, Gambari R, Cinatl J, Doerr HW (2008). Chem. Biodivers..

[18] Chen CJ, Michaelis M, Hsu HK, Tsai CC, Yang KD, Wu YC, Cinatl J, Doerr HW (2008). J. Ethnopharmacol..

[19] Zhuang M, Jiang H, Suzuki Y, Li X, Xiao P, Tanaka T, Ling H, Yang B, Saitoh H, Zhang L, Qin C, Sugamura K, Hattori T (2009). Antiviral Res..

[20] Luo W, Su X, Gong S, Qin Y, Liu W, Li J, Yu H, Xu Q (2009). Biosci. Trends.

[21] Yook HS, Kim KH, Park JE, Shin HJ (2010). Am. J. Chin. Med..

[22] Ulasli M, Gurses SA, Bayraktar R, Yumrutas O, Oztuzcu S, Igci M, Igci YZ, Cakmak EA, Arslan A (2014). Mol. Biol. Rep..

[23] Chen C, Zuckerman DM, Brantley S, Sharpe M, Childress K, Hoiczyk E, Pendleton AR (2014). BMC Vet. Res..

[24] Lelešius R, Karpovaitė A, Mickienė R, Drevinskas T, Tiso N, Ragažinskienė O, Kubilienė L, Maruška A, Šalomskas A (2019). BMC Vet. Res..

[25] Fiore C, Eisenhut M, Krausse R, Ragazzi E, Pellati D, Armanini D, Bielenberg J (2005). Phytother. Res..

[26] Cinatl J, Morgenstern B, Bauer G, Chandra P, Rabenau H, Doerr HW (2003). Lancet.

[27] Wu CY, Jan JT, Ma SH, Kuo CJ, Juan HF, Cheng YS, Hsu HH, Huang HC, Wu D, Brik A, Liang FS, Liu RS, Fang JM, Chen ST, Liang PH, Wong CH (2004). Proc. Natl. Acad. Sci. USA.

[28] Kim JW, Ha TK, Cho H, Kim E, Shim SH, Yang JL, Oh WK (2017). Bioorg. Med. Chem. Lett..

[29] Cheng PW, Ng LT, Chiang LC, Lin CC (2006). Clin. Exp. Pharmacol. Physiol..

[30] Yi L, Li Z, Yuan K, Qu X, Chen J, Wang G, Zhang H, Luo H, Zhu L, Jiang P, Chen L, Shen Y, Luo M, Zuo G, Hu J, Duan D, Nie Y, Shi X, Wang W, Han Y, Li T, Liu Y, Ding M, Deng H, Xu X (2004). J. Virol..

[31] Lin CW, Tsai FJ, Tsai CH, Lai CC, Wan L, Ho TY, Hsieh CC, Chao PD (2005). Antiviral Res..

[32] Li H, Wu J, Zhang Z, Ma Y, Liao F, Zhang Y, Wu G (2011). Phytother. Res..

[33] Ryu YB, Jeong HJ, Kim JH, Kim YM, Park JY, Kim D, Nguyen TT, Park SJ, Chang JS, Park KH, Rho MC, Lee WS (2010). Bioorg. Med. Chem..

[34] Kim DW, Seo KH, Curtis-Long MJ, Oh KY, Oh JW, Cho JK, Lee KH, Park KH (2014). J. Enzyme Inhib. Med. Chem..

[35] Cho JK, Curtis-Long MJ, Lee KH, Kim DW, Ryu HW, Yuk HJ, Park KH (2013). Bioorg. Med. Chem..

[36] Song YH, Kim DW, Curtis-Long MJ, Yuk HJ, Wang Y, Zhuang N, Lee KH, Jeon KS, Park KH (2014). Biol. Pharm. Bull..

[37] Park JY, Yuk HJ, Ryu HW, Lim SH, Kim KS, Park KH, Ryu YB, Lee WS (2017). J. Enzym. Inhib. Med. Chem..

[38] Shen YC, Wang LT, Khalil AT, Chiang LC, Chem PW (2005). Pharm. Bull..

[39] Zhang DH, Wu KL, Zhang X, Deng SQ, Peng B (2020). J. Integr. Med..

[40] Ul Qamar MT, Alqahtani SM, Alamri MA, Chen LL (2020). J. Pharm. Anal..

[41] Li SY, Chen C, Zhang HQ, Guo HY, Wang H, Wang L, Zhang X, Hua SN, Yu J, Xiao PG, Li RS, Tan X (2005). Antiviral Res..

[42] Yang CW, Lee YZ, Kang IJ, Barnard DL, Jan JT, Lin D, Huang CW, Yeh TK, Chao YS, Lee SJ (2010). Antiviral Res..

[43] Kang KB, Ming G, Kim GJ, Ha TK, Choi H, Oh WK, Sung SH (2015). Phytochemistry.

[44] Wen CC, Kuo YH, Jan JT, Liang PH, Wang SY, Liu HG, Lee CK, Chang ST, Kuo CJ, Lee SS, Hou CC, Hsiao PW, Chien SC, Shyur LF, Yang NS (2007). J. Med. Chem..

[45] Park JY, Kim JH, Kim YM, Jeong HJ, Kim DW, Park KH, Kwon HJ, Park SJ, Lee WS, Ryu YB (2012). Bioorg. Med. Chem..

[46] Chang FR, Yen CT, El-Shazly M, Lin WH, Yen MH, Lin KH, Wu YC (2012). Nat. Prod. Commun..

[47] Yang JL, Ha TK, Dhodary B, Pyo E, Nguyen NH, Cho H, Kim E, Oh WK (2015). J. Med. Chem..

[48] Liu LL, Ha TK, Ha W, Ohi WK, Yangi JL, Shi YP (2017). J. Nat. Prod..

[49] Ho TY, Wu SL, Chen JC, Li CC, Hsiang CY (2007). Antiviral Res..

[50] Hsieh LE, Lin CN, Su BL, Jan TR, Chen CM, Wang CH, Lin DS, Lin CT, Chueh LL (2010). Antiviral Res..

[51] Yang QY, Tian XY, Fang WS (2007). J. Asian Nat. Prod. Res..

[52] Park JY, Ko JA, Kim DW, Kim YM, Kwon HJ, Jeong HJ, Kim CY, Park KH, Lee WS, Ryu YB (2016). J. Enzyme Inhib. Med. Chem..

[53] Park JY, Jeong HJ, Kim JH, Kim YM, Park SJ, Kim D, Park KH, Lee WS, Ryu YB (2012). Biol. Pharm. Bull..

[54] Keyaerts E, Vijgen L, Pannecouque C, Van Damme E, Peumans W, Egberink H, Balzarini J, Van Ranst M (2007). Antiviral Res..

[55] Lee JH, Park JS, Lee SW, Hwang SY, Young BE, Choi HJ (2015). Virus Res..

[56] Zhang P, Liu X, Liu H, Wang W, Liu X, Li X, Wu X (2018). Microb. Pathog..

[57] Zhang P, Wang J, Wang W, Liu X, Liu H, Li X, Wu X (2017). Microb. Pathog..

[58] Ma X, Bi S, Wang Y, Chi X, Hu S (2019). Poult. Sci..

[59] Nguyen PQ, Ooi JS, Nguyen NT, Wang S, Huang M, Liu DX, Tam JP (2015). J. Biol. Chem..

